# Hydrogen bonding and packing density are factors most strongly connected to limiting sites of high flexibility in the 16S rRNA in the 30S ribosome

**DOI:** 10.1186/1472-6807-9-49

**Published:** 2009-07-30

**Authors:** Wayne Huggins, Sujit K Ghosh, Paul Wollenzien

**Affiliations:** 1 Department of Molecular and Structural Biochemistry, North Carolina State University, Raleigh, USA; 2 RTI International, Research Triangle Park, USA; 3 Department of Statistics, North Carolina State University, Raleigh, USA

## Abstract

**Background:**

Conformational flexibility in structured RNA frequently is critical to function. The 30S ribosomal subunit exists in different conformations in different functional states due to changes in the central part of the 16S rRNA. We are interested in evaluating the factors that might be responsible for restricting flexibility to specific parts of the 16S rRNA using biochemical data obtained from the 30S subunit in solution. This problem was approached taking advantage of the observation that there must be a high degree of conformational flexibility at sites where UV photocrosslinking occurs and a lack of flexibility inhibits photoreactivity at many other sites that are otherwise suitable for reaction.

**Results:**

We used 30S x-ray structures to quantify the properties of the nucleotide pairs at UV- and UVA-s^4^U-induced photocrosslinking sites in 16S rRNA and compared these to the properties of many hundreds of additional sites that have suitable geometry but do not undergo photocrosslinking. Five factors that might affect RNA flexibility were investigated – RNA interactions with ribosomal proteins, interactions with Mg^2+ ^ions, the presence of long-range A minor motif interactions, hydrogen bonding and the count of neighboring heavy atoms around the center of each nucleobase to estimate the neighbor packing density. The two factors that are very different in the unreactive inflexible pairs compared to the reactive ones are the average number of hydrogen bonds and the average value for the number of neighboring atoms. In both cases, these factors are greater for the unreactive nucleotide pairs at a statistically very significant level.

**Conclusion:**

The greater extent of hydrogen bonding and neighbor atom density in the unreactive nucleotide pairs is consistent with reduced flexibility at a majority of the unreactive sites. The reactive photocrosslinking sites are clustered in the 30S subunit and this indicates nonuniform patterns of hydrogen bonding and packing density in the 16S rRNA tertiary structure. Because this analysis addresses inter-nucleotide distances and geometry between nucleotides distant in the primary sequence, the results indicate regional and global flexibility of the rRNA.

## Background

There is a long-standing interest in the connection between the interior packing arrangement and conformational dynamics of proteins. Richards [1] recognized early the importance of packing in the protein interior and summarized methods for calculating packing density; he further speculated that irregular packing, if it results in gaps or cavities, could lead to specific conformational motions. This idea lacked support for some time, given evidence that efficient packing was important to protein stability and rapid folding [2-4]. More recently Liang and Dill [5] used several parameters to measure the distribution of free volumes in proteins and concluded that many proteins appeared to be packed in ways that result in heterogeneous environments and have significant frequencies of packing defects. In addition, a model for calculating conformational dynamics, the Gaussian Network Model, has been used successfully to account for local motions [6]. One conclusion from that work is that the packing density at each amino acid residue plays a major role, at least on the intermediate time scale, in determining local vibrational motions [7].

Nucleic acids are also likely to exhibit similar connections between flexibility and structure taking in account differences in the size, shape and charge in the nucleotide units compared to the amino acids. This problem has not been addressed to the same extent as in proteins, probably due the lack of high resolution structures. However advances in crystallography in the last ten years have provided a larger variety of, and larger-sized, RNA and DNA structures at atomic resolution [8]. The ribosome is a striking example of a large RNA-protein complex whose detailed structure has been successfully solved by x-ray crystallography [9-13]. For both of the ribosomal subunits, the structures are defined by the compactly folded ribosomal RNA in which intramolecular interactions, including helix stacking and numerous structural motifs stabilize the global arrangement [14,15]. At the same time, the issue of the intrinsic conformational flexibility in the ribosome is important because it undergoes specific conformational changes that control tRNA and mRNA association and movement [16-18]. This problem has been addressed computationally by Tama *et al*. [19] and Wang *et al*. [20] who used Elastic Network Models to determine intrinsic motions. Both groups found the lowest modes of vibration which indicate the largest and slowest conformational motions to be related to the conformational changes observed by cryo electron microscopy in different functional states.

Several analyses have been done to interpret biochemical data pertaining to flexibility in the ribosome in light of the crystal structures. Differences between RNA-RNA and RNA-protein distances derived from experimental data including chemical and hydroxyl radical foot-printing, crosslinking with different reagents and accessibility data and corresponding distances calculated from the x-ray structures were used to identify sites where there is conformational flexibility or alternative conformations [21,22]. About one fourth of calculated distances, after removal of measurements that must come from experimental error and allowing for uncertainty, were considered discrepant because they indicate larger or smaller distances than seen in the x-ray structure. Importantly, in both ribosome subunits, the discrepant measurements are clustered in a restricted part of the subunit and are self consistent, leading to the conclusion that these mismatches indicate specific types of internal conformational motions [23]. In the 30S subunit, the data are consistent with movements that close or open the 30S structure around the decoding region; in the 50S subunit, the data indicate movements of peripheral regions surrounding the upper central part around the central protuberance [23].

The underlying factors that allow flexibility in specific regions, but not in others, have not been identified for either ribosomal subunit. Consequently we have utilized RNA-RNA photocrosslinking data that are available for the 16S rRNA to investigate this question. The pattern of UV crosslinking is determined by features related to the 30S tertiary structure rather than by photochemical factors [24-26]. This is likely to involve flexibility because the RNA-RNA photocrosslinks occur in the same region where other biochemical data indicate conformational flexibility [23,26]. In support of flexibility as the critical factor is the conclusion that RNA-RNA photocrosslinking is under thermodynamic control. In general nucleotide pairs that are closer together in the crystal structures have higher crosslinking frequencies for both UV and UVA-s^4^U photocrosslinking and this is consistent with the photocrosslinking mechanism involving transient nucleotide displacements from equilibrium positions rather than involving stable alternative local conformations different than the conformations present in the crystal structure [26]. The average internucleotide distances measured between photoreactive bonds for the UV and UVA-s^4^U reactive sites are 7.5 Å and 10.9 Å, so most of these displacements are much larger than would normally occur in the RNA through thermal motions. Other sites that are unreactive despite having suitable internucleotide arrangements must not be capable of the displacements needed for photocrosslinking and are considered inflexible with respect to these motions.

We have compared the properties of the photoreactive nucleotide pairs to the properties of unreactive nucleotide pairs to gain insight into factors that allow or inhibit flexibility. The properties that are considered are RNA-protein interactions, RNA packing density, Mg^2+ ^binding, long-range RNA interactions through the A minor motif, and hydrogen bonding. The factors that are important should be seen for both types of photocrosslinking reactions and should be seen using data from different x-ray structures. We conclude that the factors that are different at a statistically significant level between the reactive and unreactive sites are hydrogen bonding and the packing density.

## Results

### Identification of potential 16S rRNA UV and UVA-s^4^U crosslinking sites in different 30S structures

**Figure 1:**
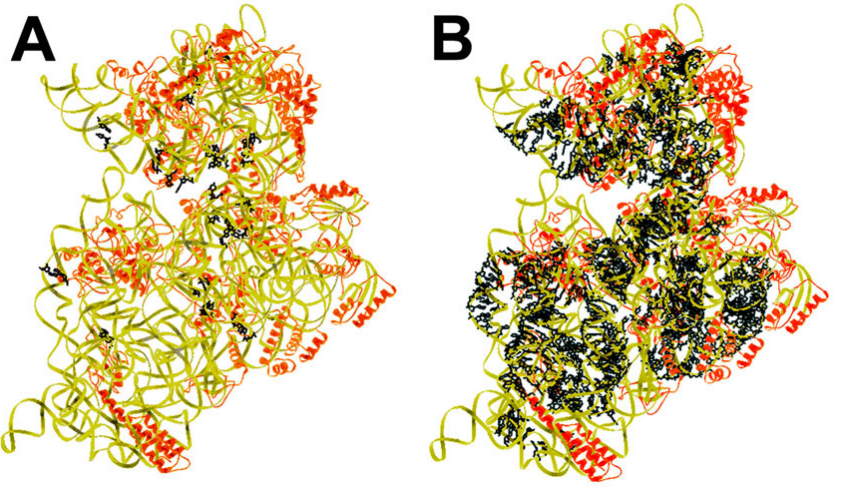
**Locations of reactive and unreactive sites for UV-induced photocrosslinking in the 30S structure**. A. Sites of observed UV-induced photocrosslinks. The *E. coli *II structure [13] is used for the figure. The orientation of the 30S subunit in this and the subsequent figures is of the subunit interface side facing the viewer. The 30S head is upwards. RNA and proteins are represented with yellow and orange ribbons, respectively. The nucleotides involved in photocrosslinking are drawn in black. B. Sites of potential but unreactive sites for UV-induced photocrosslinking. The figure contains the 714 nucleotide pairs found in the *E. coli *II structure that should be suitable for reaction. The figures were prepared with the program Ribbons [52].

**Figure 2:**
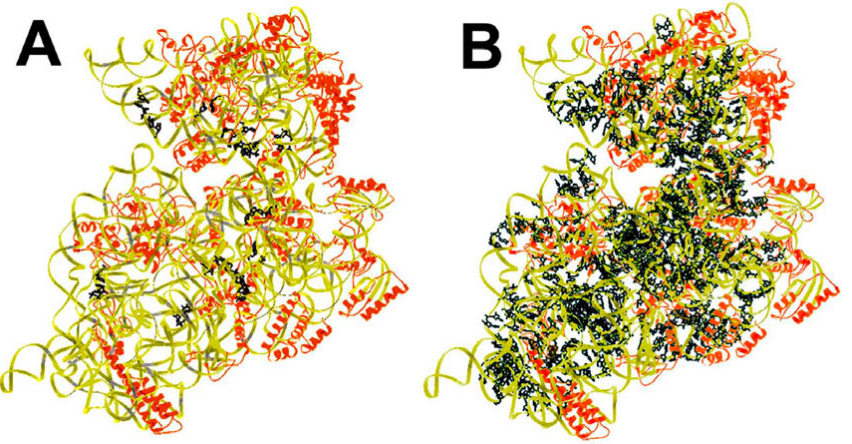
**Locations of reactive and unreactive sites for UVA-s^4^U-induced photocrosslinking sites in the 30S structure**. A. Sites of observed UVA-s^4^U-induced photocrosslinks. Nucleotides involved in photocrosslinking are drawn in black. B. Sites of potential but unreactive sites for UVA-s^4^U photocrosslinking. The figure indicates the sites of the 940 nucleotide pairs found in the *E. coli *II structure that should be suitable for reaction based on internucleotide geometry.

**Figure 3:**
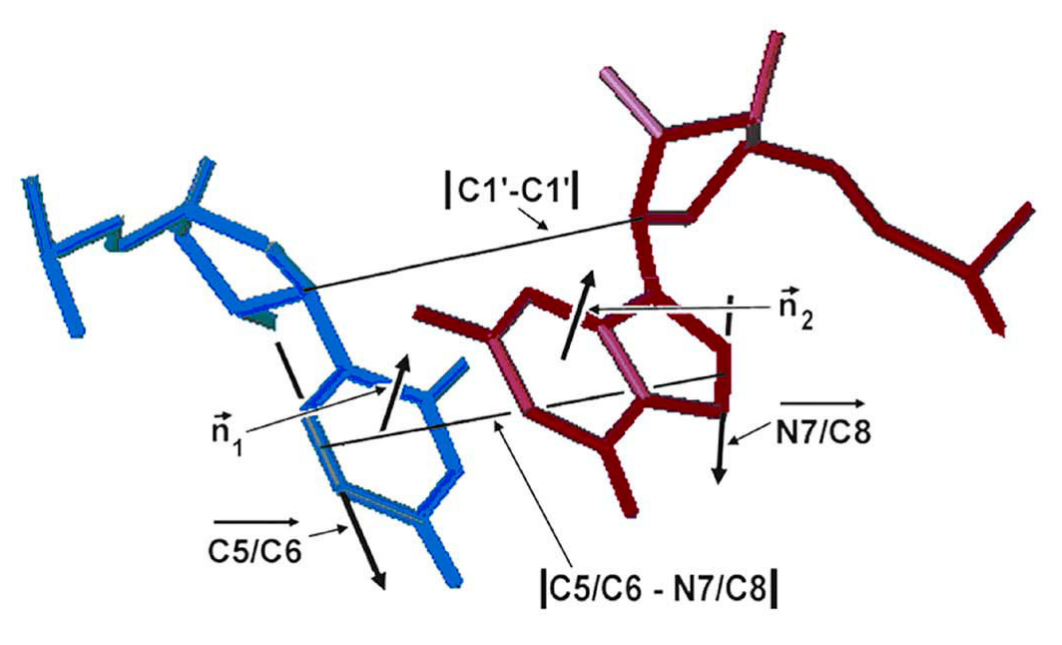
**Internucleotide geometrical parameters. Distance and geometry measurements for a nucleotide pair are illustrated on 16S rRNA nucleotides U244 and G894 which form a UV-induced photocrosslink**. The distances between reactive bonds (double bonds at C5/C6 and N7/C8 in this instance), and C1'-C1' are indicated. The angle between respective base planes, here called base plane angle, is calculated as the angle between the perpendicular vectors (n_1 _and n_2_) to the planes defined by the C2, C4 and C6 atoms of the pyrimidine or purine. The angle, here called the reactive bond angle, is between the vectors defined by the C5/C6 atoms of the pyrimidine and the N7/C8 atoms of the purine.

The locations of the intramolecular crosslinks in the 16S rRNA produced by irradiation with UV light [27,28] or by UVA irradiation of ribosomes containing s^4^U [25] are shown in Figure 1A and Figure 2A. The properties of these sites were used to identify additional places in the RNA structure that should be photoreactive by ranking potential sites according to their hypothetical crosslinking frequencies. The details of the photoreactions are known for both types of photocrosslinking, as described in the Methods section, so features that could be related to photocrosslinking frequency were calculated from the *T. thermophilus *30S subunit x-ray structure [11] and from two *E. coli *30S subunit x-ray structures [13]. The features included the distances and angles between the photoreactive bonds, other features of the internucleotide geometry (Figure 3) and the crystallographic B factors, indicators of thermal motions. The largest and most reproducible correlation involving the crosslinking frequencies was observed with the inverse of the C1'-C1' internucleotide distances (Table 1). In addition, correlations were present between the angles between the nucleobase planes and the distances between the photoreactive bonds (Table 1). Correlations between frequencies and distance between reactive bonds or between frequencies and angles between reactive bonds were more variable using data from the different x-ray structures [see Additional file 1], probably due to larger differences in nucleobase positioning in the different structures.

**Table 1:** Correlation coefficients between crosslinking frequencies and internucleotide geometry in different 30S structures

	**Measure^1^**	***E. coli *I**	***E. coli *II**	***T. thermophilus***	**Ave.**
**UVB**	Freq. vs 1/(C1'-C1')	0.627	0.495	0.389	0.504
	Freq. vs B factor	0.464	0.402	0.296	0.386
					
**UVA-s^4^U**	Freq. vs 1/(C1'-C1')	0.388	0.389	0.538	0.438
	Freq. vs B factor	0.567	0.317	0.269	0.384
					
**UVB**	BPA vs RBD	0.410	0.447	0.555	0.471
					
**UVA-s^4^U**	BPA vs RBD	0.394	0.366	0.783	0.514

^1^Measures are: C1'-C1', distance between C1' atoms; RBD, distance between centers of reactive bonds; RBA, angle between the reactive bonds; BPA, angle between the planes of the bases. The B factor is the average calculated for the heavy atoms of the nucleobases in each nucleotide pair. The correlation coefficient is the Pearson product-moment correlation coefficient. The geometrical parameters were calculated from the *E. coli *I, *E. coli *II [13] and *T. thermophilus *[11] 30S structures.

Regression analysis also was used to relate internucleotide geometry and photocrosslinking frequencies. Regression equations that connect crosslinking frequencies to inverse C1'-C1' distances or to both internucleotide distance and angle in a non-linear equation are statistically better than equations that use additional geometrical parameters (Table 2). The adjusted R^2 ^values for these equations indicate that differences in the internucleotide geometry account for 54% to 70% of the variance of the photocrosslinking frequencies.

**Table 2:** Linear regression models and statistics for estimating crosslinking frequencies


a) Estimation of UV crosslinking frequencies from internucleotide geometry^1^				
**Model:Freq = β _1_(1/(C1'-C1'))**			**Statistics^2^**	
Data – Variables	β_1_		Adj. R^2^	p-value
1a – 1/(C1'-C1')	2.66		0.69	5.6 × 10^-6^
1b – 1/(C1'-C1')	2.58		0.64	2.2 × 10^-5^
1c – 1/(C1'-C1')	2.80		0.60	5.6 × 10^-5^
**Model:Freq. = β_1_(1/(C1'-C1'))(COS(BPA)) + β_2_(1/(C1'-C1'))(SIN(BPA))**				
Data – Variables	β_1_	β_2_	Adj. R^2^	p-value
2a – 1/(C1'-C1'), BPA	1.4	3.0	0.69	3.4 × 10^-5^
2b – 1/(C1'-C1'), BPA	2.9	2.4	0.66	7.0 × 10^-5^
2c – 1/(C1'-C1'), BPA	2.9	1.2	0.61	1.9 × 10^-4^


b) Estimation of UVA-s^4^U crosslinking frequencies from internucleotide geometry^1^				

**Model:Freq = β_1_(1/(C1'-C1'))**				
Data – Variables	β_1_		Adj. R^2^	p-value
3a – 1/(C1'-C1')	11.2		0.57	7.2 × 10^-5^
3b – 1/(C1'-C1')	10.6		0.54	1.3 × 10^-4^
3c – 1/(C1'-C1')	12.0		0.62	7.2 × 10^-6^
**Model:Freq. = β_1_(1/(C1'-C1'))(COS(BPA)) + β_2_(1/(C1'-C1'))(SIN(BPA))**				
Data – Variables	β_1_	β_2_	Adj. R^2^	p-value
4a – 1/(C1'-C1'), BPA	11.9	2.6	0.54	5.3 × 10^-4^
4b – 1/(C1'-C1'), BPA	13.2	1.4	0.58	2.4 × 10^-4^
4c – 1/(C1'-C1'), BPA	8.4	11.4	0.61	4.8 × 10^-5^

^1 ^In each case, the geometrical parameters were calculated from the (a) *E. coli *I, (b) *E. coli *II [13] and (c) *T. thermophilus *[11] 30S structures. β_1 _and β_2 _are the constants determined in the linear regression analysis. C1'-C1' is the distance between C1' atoms of the nucleotides in the observed photocrosslinks. BPA is base plane angle, the torsion angle between the planes of the bases of nucleotides observed in crosslinks, measured in degrees.

^2 ^The Adj. R^2 ^values indicate the fraction of the variance of the frequency that is accounted for by the regression function. The p values are the probabilities that the frequency is not correlated to the geometry parameter(s).

Nucleotide pairs that should be sites for UV crosslinking were identified based on their internucleotide geometries and other criteria as described in the Methods section. The initial lists of nucleotide pairs consisted of pairs that were separated by not more than 10.5 Å measured between the chemical bonds that would be involved in the photochemical reaction. The 10.5 Å value was chosen because the internucleotide distances for the majority of the observed reactive nucleotide pairs fall within this range. Potential UV crosslinked nucleotide pairs were then ranked by the crosslinking frequencies predicted by their internucleotide geometries using the regression equations; nucleotide pairs with predicted frequencies at least as good as the observed photocrosslinks were retained. There are 674, 714, and 768 nucleotide pairs in the *E. coli *I, *E. coli *II and *T. thermophilus *structures that are favorable for crosslinking using these criteria. The distribution of the sites is shown in Figure 1B.

Nucleotide pairs that should be sites for UVA-s^4^U-induced crosslinking were identified using similar methods. In this case, an 18 Å cut-off value was picked to include the majority of the reactive UVA-s^4^U sites. In the 16S rRNA 118 uridines are thiolated by *in vivo *incorporation of s^4^U into the 16S rRNA [25] and these were used to predict potential crosslinking sites to insure a fair comparison of reactive and unreactive sites. Potential UVA-s^4^U crosslinking sites were ranked and retained according to the frequencies calculated from the regression equations. These data indicate 928, 940 and 893 nucleotide pairs in the *E. coli *I, *E. coli *II and *T. thermophilus *structures that have geometrical properties apparently as good as the majority of the observed crosslinking sites. The distribution of these sites is shown in Figure 2B.

To determine if there were differences in the stereochemical arrangements in the nucleotide pairs at unreactive and reactive sites, we examined selected unreactive nucleotide pairs and compared these to the photoreactive nucleotide pairs. The unreactive pairs were picked to match the internucleotide reactive bond distances of the observed photocrosslinked sites, but otherwise were picked randomly. The properties of the potentially crosslinked and observed crosslinked nucleotide pairs were quantitatively similar [see Additional file 1]. However, for 31 potential UV crosslinked nucleotide pairs that were inspected, there were ten pairs in which the nucleobases crossed over each other or were farther apart in the direction perpendicular to the nucleobase plane than seen in the photoreactive nucleotide pairs. For 32 potentially crosslinked UVA-s^4^U nucleotide pairs, ten were in arrangements in which the nucleobases were farther away from each other in the direction perpendicular to the nucleobase than was seen in the reactive nucleotide pairs. Taken together, these results indicate that there are about 450 to 510 nucleotide pairs and about 590 to 630 nucleotide pairs that are unreactive for UV and UVA-s^4^U photocrosslinking, respectively, in each of the three x-ray structures, even though they have arrangements suitable for reaction.

### B factors are smaller in the inflexible unreactive nucleotide pairs

The B factors for the unreactive nucleotide pairs are somewhat smaller than those in the reactive nucleotide pairs. For UV crosslinking, the z value of 1.50 that was calculated for the difference in the average of the B factor values is less than the two-sided 5% cut-off value of 1.96 needed to conclude that the difference is significant at the 5% level. It should be noted that the standard deviations in these values and in the other evaluated structural parameters are quite large, reflecting the heterogeneity in the geometries and properties at the different nucleotide pairs in both the reactive and unreactive sites. However, because of the number of measurements, the standard errors of the mean are much smaller than the standard deviations and allow conclusions about the significance of the differences between the averages of the reactive and unreactive nucleotide pairs. A second comparison to determine if the reactivity is due to the more flexible of the two nucleotides in each pair was done by picking the larger of the average B factors for each nucleotide pair for comparison. The differences in the averages of these values are somewhat larger (Table 3); however, the z value of 1.90 is still a little less than the value 1.96 needed for the conclusion that the difference is significant at the 5% level.

**Table 3:** Comparison of B factors, hydrogen bonding and neighbor atom count around reactive and unreactive nucleotide pairs

**UV reactions**					Statistics^3^	
	Nucleotide Pairs Reactive		Unreactive			
Parameter	n^1^	Ave. ± S.D.	n^2^	Ave. ± S.D.	z value	p value
B factor for both nt.	42	65.8 ± 26.8	2156	58.0 ± 26.1	1.917	0.0553
Value of larger B factor of pair	42	77.8 ± 36.1	2156	67.3 ± 28.0	2.392	0.0168
H bonds/nt. for both nt.	42	1.13 ± 1.2	523	1.70 ± 0.85	-4.038	< 0.0001
H bonds in lesser H-bonded nt.	42	0.47 ± 0.73	523	0.97 ± 1.04	-3.055	0.0024
Atom count around both nt.	42	21.8 ± 8.9	2156	28.0 ± 9.3	-4.282	< 0.0001
Atom count – lower-packed nt.	42	15.6 ± 11.0	2156	23.2 ± 9.5	-5.249	< 0.0001

**UVA-s^4^U reactions**					Statistics	
	Nucleotide Pairs Reactive		Unreactive			

Parameter	n^4^	Ave. ± S.D.	n^5^	Ave. ± S.D.	z value	p value
B factor for both nt.	50	66.3 ± 24.9	2761	58.5 ± 28.6	1.915	0.0556
Value of larger B factor of pair	50	74.1 ± 25.7	2761	68.8 ± 31.2	1.194	0.2327
Value of s^4^U B factor	24	72.0 ± 25.4	315	66.3 ± 31.9	0.855	0.3934
H bonds/nt. for both nt.	50	1.27 ± 0.90	844	1.76 ± 0.77	-4.329	< 0.0001
H bonds/s^4^U	24	0.71 ± 0.91	315	1.55 ± 0.96	-4.146	< 0.0001
Atom count around both nt.	50	25.6 ± 11.3	2761	30.8 ± 8.4	-4.431	< 0.0001
Atom count around the s^4^U	24	21.0 ± 11.6	315	30.0 ± 8.8	-4.712	< 0.0001

^1^The number of reactive nucleotide pairs evaluated is 14, 13 and 15 in the *T. thermophilus *[11], *E. coli *I and *E. coli *II [13] structures respectively, after removal of observed crosslinks that have > 10.5 Å between reactive bonds and because two crosslinking sites are not present in the *T. thermophilus *structure. The average and standard deviations here, and in other data, are weighted averages and standard deviations from three sets of measurements.

^2^The number of measurements of unreactive pairs is 768, 674 and 714 in the *T. thermophilus*, *E. coli *I and *E. coli *II structures respectively, except for the hydrogen bonding frequencies which were evaluated from a representative number of nucleotide pairs in each structure.

^3 ^z-value is the difference of sample means normalized by the standard error of the means. The null hypothesis, that the populations have the same averages, can be rejected at the 5% and 1% level of significance if |z| ≥ 1.96 and |z| ≥ 2.56, respectively. p-value is the probability that random sampling would lead to a difference between sample means as large (or larger) than that has been observed, if the null hypothesis is true, i.e., if the populations have the same true mean.

^4^The number of reactive nucleotide pairs evaluated is 18, 16 and 16 in the *T. thermophilus*, *E. coli *I and *E. coli *II structures, after removal of observed crosslinks that have > 18 Å between reactive bonds. For evaluation of H bonds/s^4^U and atom count around the s^4^U, there are eight s^4^Us that are involved in the 18 photocrosslinks that are evaluated in each of the three structures.

^5^The number of measurements of unreactive pairs is 893, 928 and 940 in the *T. thermophilus*, *E. coli *I and *E. coli *II structures respectively, except for the hydrogen bonding frequencies which were evaluated manually on a representative number of nucleotide pairs in each structure and for the value of the s^4^U B factor, H bonds/s^4^U, and atom count around the s^4^U which were evaluated just once on each of the 105 substituted s^4^U positions in each structure.

Comparisons of average B factors also were done for the potential and observed UVA-s^4^U photocrosslinking sites. The comparisons were of the average B factor for both nucleotides in each pair, of the B factor for the s^4^U in each pair and of the nucleotide of each pair with the higher B factor. The differences in the averages again indicate that the unreactive nucleotide pairs have somewhat smaller B factors than the reactive pairs (Table 3), again consistent with a connection between conformational flexibility and reactivity. Differences for the s^4^U comparisons were less than for the UV comparisons. We conclude that B factors are generally smaller for the unreactive nucleotide pairs but not at a statistically significant value.

### RNA-protein interactions are similar at the unreactive and reactive nucleotide pairs

The 30S higher order structure contains protein-nucleotide interactions that could be a factor in reducing the conformational flexibility at the unreactive potential sites. Brodersen *et al*. [29] identified 513 nucleotides (of the 1521 nucleotides in the *T. thermophilus *16S rRNA) that are within 3.5 Å of any part of the ribosomal proteins. If contacts involving only the nucleobase part of the nucleotides are considered, there are 178 protein contacts.

The fraction of the nucleotides involved in nucleobase-protein interactions for photoreactive nucleotide pairs is 0.14 and 0.26 for the UV and UVA-s^4^U crosslinks, respectively. These values are larger, but not significantly, than the value of 0.12 for the nucleotides in the unreactive nucleotide pairs. The fraction of the crosslinked nucleotides involved in interactions with proteins involving any part of the nucleotide is 0.31 and 0.57 for the UVB and UVA-s^4^U crosslinked nucleotides compared to 0.34 for the nucleotides in the unreactive nucleotide pairs, again larger for the reactive pairs but not significantly. Therefore these values for the crosslinked nucleotides are overall larger than expected on a random basis but not at a significant level. In addition, the majority of the crosslinked nucleotide pairs (12 pairs in each type of crosslinking) do not have contacts with proteins, so contacts with proteins cannot be a prerequisite for crosslinking and it is unlikely that there is a general connection between flexibility and protein contacts.

### Differences in the frequency of A-minor motif RNA contacts at the unreactive versus reactive sites are not significant

Stable RNA tertiary structure interactions could be the reason for restricted flexibility at some sites within the RNA. The A minor motif is the most common tertiary structure motif. This motif involves interactions between adenosines and helical receptors mediated by hydrogen bonding between the adenosine and nucleotides of the receptor in the helix minor groove [30]. In the *T. thermophilus *16S rRNA structure there are 55 instances of A-minor motif contacts at 31 sites or in positions very close to the correct arrangement [31].

None of the observed UV-induced photocrosslinked sites are involved in A-minor motif contacts, and of the 714 and 674 nucleotide pairs that are potential UV-induced crosslinking sites in the *T. thermophilus *and *E. coli *II 16S rRNA, 16 and 15 pairs, respectively, are involved in A-minor motif interactions. Similarly, none of the UVA-s^4^U-induced photocrosslinking sites are involved in A-minor motif interactions, and only 4 and 5 pairs, respectively, of the 940 and 928 nucleotide pair potential UVA-s^4^U induced crosslinking sites in the *T. thermophilus *and *E. coli *II 16S rRNA are involved in A-minor motif interactions. For both types of crosslinking sites, there is no difference at the 5% level of significance in the frequencies of reactive pairs or unreactive pairs in the A-minor motif interactions (Table 4). Furthermore, only a small fraction of the total number of unreactive nucleotide pairs are involved in the A minor structure.

**Table 4:** Comparison of frequency of A minor motif interactions and Mg^2+ ^binding in the vicinity of the reactive and unreactive nucleotide pairs

**UV reactions**	Nucleotide Pairs		Statistics^1^	
Parameter	Reactive	Unreactive	z value	p value
Number of nt. pairs in A minor motif^2^	0 of 14 nt. pairs	31 of 1388 nt. pairs	-0.5998	0.549
Mg^2+ ^in vicinity of nt.^3^	54 Mg^2+ ^at 56 nt.	613 Mg^2+ ^at 698 nt.	0.2324	0.816
Mg^2+ ^bridges in vicinity of nt. pair^4^	2 of 28 nt. pairs	131 of 4312 nt. pairs	1.1695	0.242
				
**UVA-s^4^U reactions**	Nucleotide Pairs		Statistics	
Parameter	Reactive	Unreactive	z value	p value
Number of nt. pairs in A minor motif^5^	0 of 18 nt. pairs	9 of 1868 nt. pairs	-0.3007	0.764
Mg^2+ ^in vicinity of nt.^6^	31 Mg^2+ ^at 54 nt.	3034 Mg^2+ ^at 3660 nt.	-0.9186	0.359
Mg^2+ ^bridges in vicinity of nt. pair^7^	1 of 36 nt. pairs	71 of 5522 nt. pairs	0.7528	0.452

^1 ^z-value is the difference of sample means normalized by the standard error of the means. The null hypothesis, that the populations have the same averages, can be rejected at the 5% and 1% level of significance if |z| ≥ 1.96 and |z| ≥ 2.56, respectively. p-value is the probability that random sampling would lead to a difference between sample means as large (or larger) than that has been observed, if the null hypothesis is true, i.e., if the populations have the same true mean.

^2^Sixteen nucleotide pairs in observed crosslinks and 671 and 712 nucleotide pairs at unreactive sites in the *E. coli *I and II structures [13] were evaluated using the list of A minor motif interactions [31].

^3^Twenty nine nucleotides from the observed UV crosslinks and 698 randomly selected nucleotides from the list of unreactive nucleotide pairs in the *T. thermophilus *[11] and *E. coli *I and II structures [13] were evaluated using the lists of Mg^2+ ^interaction in the *T. thermophilus *I and II structures [34].

^4^Fifteen nucleotide pairs in observed crosslinks and 2156 nucleotide pairs at unreactive sites in the *T. thermophilus *[11] and *E. coli *I and II [13] structures were evaluated using the lists of the Mg^2+ ^interactions sites in the *T. thermophilus *I and II structures [34].

^5^Eighteen nucleotide pairs in observed crosslinks and 928 and 940 nucleotide pairs at unreactive sites in the *E. coli *I and II structures [13] were evaluated using the list of A minor motif interactions [31].

^6^Twenty seven nucleotides from the observed UVA-s^4^U crosslinks and 3660 randomly selected nucleotides from the list of unreactive nucleotide pairs in the *T. thermophilus *[11] and *E. coli *I and II structures [13] were evaluated using the lists of Mg^2+ ^interaction in the *T. thermophilus *I and II structures [34].

^7^Eighteen nucleotide pairs in observed crosslinks and 2761 nucleotide pairs at unreactive sites in the *T. thermophilus *[11] and *E. coli *I and II [13] structures were evaluated using the lists of the Mg^2+ ^interactions sites in the *T. thermophilus *I and II structures [34].

### Mg^2+ ^binding at the interacting nucleotide pairs does not account for the differences in the unreactive and reactive sites

The correct level of Mg^2+ ^is necessary for optimal ribosome function [32,33], consistent with the need for stabilization of the native structure by Mg^2+^. Mg^2+ ^ions bridges between nucleotides could be associated with inhibition of internucleotide flexibility so this was investigated as a possible difference between reactive and unreactive nucleotide pairs. Interactions between Mg^2+ ^and nucleotide atoms were listed in two *T. thermophilus *30S subunit structures reported by Selmer *et al*. [34] allowing determination of the frequencies of Mg^2+ ^binding and of Mg^2+^-mediated bridges in the vicinity of the unreactive and reactive nucleotide pairs.

Interactions with Mg^2+ ^within a five-nucleotide interval (Table 4) were tabulated for nucleotides in the reactive and unreactive nucleotide pairs. The differences in the frequencies of the nucleotides associated with Mg^2+ ^in the reactive and unreactive sites are not significant for either the UV or the UVA-s^4^U sites. In addition, the frequencies of nucleotide pairs in the vicinity of Mg^2+^-mediated bridges at the reactive and unreactive sites were compared and were found also to be not statistically different (Table 4). Importantly, the number of nucleotide pairs that could be affected by the Mg^2+^-mediated bridges is only a small fraction of the potentially photoreactive nucleotide pairs in the structure.

### Hydrogen bonding is greater on average at the inflexible unreactive nucleotide pairs compared to the reactive nucleotide pairs

During manual inspection of potential crosslinking sites, it was seen that there were many instances where potential photocrosslinking sites were involved in hydrogen bonding interactions. These might inhibit their movement and explain their lack of reactivity. This possibility was investigated by determining if there are differences in the average number of hydrogen bonds in the unreactive and reactive nucleotide pairs. Nucleotide pairs involved in base pairing with each other or within regular base-paired regions were removed from consideration at the initial step in listing potential sites, but these criteria did not remove pairs that are base-paired to third party nucleotides.

Hydrogen bonds in both secondary structure and tertiary structure interactions were counted. The secondary structure diagram [35] was used to list hydrogen bonding due to the secondary structure interactions. For hydrogen bonding due to tertiary structure interactions, an algorithm was developed to identify hydrogen bonds having the right geometry as well as the right distance between hydrogen bond donor and acceptor pairs [36,37] (see Methods section). This algorithm identified 318 hydrogen bonds involving 217 nucleotides in the *T. thermophilus *tertiary structure. A very similar list subsequently was found using the program HBexplore [38]. HBexplore was used to analyze the tertiary hydrogen bonds in the *E. coli *I and II structures.

Two analyses were made to compare hydrogen bonding in the UV reactive and unreactive nucleotide pairs. First, the total number of hydrogen bonds per nucleotide was determined. The average number of hydrogen bonds per nucleotide is smaller for the nucleotides in the reactive sites compared to the nucleotides in the unreactive sites at the 1% significance level (Table 3). Second, a comparison was made to determine if there would be a difference the average hydrogen bonding in the lesser hydrogen-bonded nucleotide of each pair in the reactive and unreactive nucleotide pairs. The difference in the values is also statistically significant at the 1% level (Table 3).

For the UVA-s^4^U reactive and unreactive nucleotide pairs, first the average hydrogen bonding per nucleotide for both nucleotides was compared (Table 3). Second, the average hydrogen bonding for the s^4^U in each reactive and unreactive pair was compared (Table 3). Both of these comparisons show much lower levels of hydrogen bonding in the reactive nucleotide pairs compared to the unreactive nucleotide pairs and both differences are significant at the 1% level (Table 3).

The hydrogen bonding differences found for both types of crosslinking might be explained by the fact that a larger fraction of nucleotide pairs in the unreactive sites compared to the reactive sites contained one of the partners in a double-stranded region. This would increase the measurement of hydrogen bonding because of high values for hydrogen bonding in double-stranded regions. However, comparison of the expected values, which take into account the fraction of the pairs with one single-stranded and one double-stranded nucleotide and the measured values, shows that the unreactive sites have even higher measured values of hydrogen bonding than are expected [see Additional file 1]. For instance for hydrogen bonding in the UV unreactive sites, the average number of hydrogen bonds expected is 0.98 ± 1.25, based on the fraction of the nucleotide pairs involving a nucleotide in a single-stranded region with a nucleotide in a double-stranded region, but the average number measured is 1.70 ± 0.85. The difference between the expected values and measured values for the reactive sites is smaller, 0.81 ± 1.14 and 1.12 ± 0.91. A similar larger value for the measured value compared to the expected value is seen for the unreactive and reactive UVA-s^4^U sites [see Additional file 1].

### The neighbor atom count around the unreactive nucleotide pairs is greater compared to the reactive nucleotide pairs

Another factor that might inhibit nucleotide movement at the inflexible unreactive sites is the molecular packing around each nucleotide pair. This was investigated by calculating the number of heavy atoms within a given distance of the center of each nucleobase, which should reflect the presence of stacked or intruding nucleotides in the vicinity of the nucleotide pair. This atom count does not include the atoms from the nucleotides that are the potential crosslinking partners, so it reflects close third-party nucleotides that could affect the interactions between the two potentially crosslinking partners. A counting method [7] rather than a method to calculate volume [1] was used for simplicity and because it avoided the complication of how to exclude the presence of the interacting partner nucleotide. Values for neighbor density expressed as number of heavy atom numbers within six Å from a pseudoatom in the center of each base were used. Similar data were obtained when atom counts at radii of five or seven Å were used (data not shown).

Differences in the neighbor atom count were seen when reactive and unreactive nucleotide pairs for both UV and UVA-s^4^U crosslinking were compared in two ways. In the first, the neighbor atom count for both nucleotides was considered; the values for the unreactive nucleotide pairs are larger at statistically very significant levels compared to the values for the reactive nucleotide pairs for both types of photocrosslinking sites (Table 3). In additional comparisons, the neighbor atom count for the nucleotide of each pair with the lower count value for the UV sites, or for the s^4^U of the UVA-s^4^U sites, were compared for the reactive and unreactive sites. The differences again are very significant (Table 3). It is also remarkable that the average values of neighbor atom count for the unreactive nucleotide pairs, 28.0 ± 9.3 and 30.8 ± 8.4 for the potential UV and s^4^U crosslinking sites, have values close to the values seen for nucleotides in base pairs in helical regions, 28.7 ± 3.9 for both nucleotides and 31.3 ± 5.4 for uridine residues identified as s^4^U substituted. In contrast to this, for the reactive sites for UV and s^4^U photocrosslinking, the values of the average neighbor atom counts are 22.0 ± 10.2 and 25.6 ± 11.4, respectively.

An explanation for these differences could be that a larger fraction of nucleotide pairs in the unreactive sites compared to the reactive sites contained one of the partners in a double stranded region. However, comparisons of the measured values and the expected values of neighbor atom count, which take into account the fraction of the pairs that have two single-stranded nucleotides or have a single-stranded and double-stranded nucleotide, show that the unreactive sites have even higher values of hydrogen bonding and neighbor atom count than expected [see Additional file 1].

### Comparisons of the properties of reactive and unreactive nucleotide pairs selected at different internucleotide distance cut-off values

For all of the comparisons described so far, reactive and unreactive nucleotide pairs were considered if their internucleotide distances were within 10.5 Å for the UV sites and 18 Å for the UVA-s^4^U sites. To determine if selection of the nucleotide pairs at shorter internucleotide distances would change the conclusions the analyses were repeated with data selected at shorter cut-off distance values. For the potential UV sites maximum internucleotide distances of 9 Å and 8 Å limits the number of potential unreactive sites to an average of 284 for the 9 Å cut-off value and an average of 140 for the 8 Å cut-off value in the three structures. For the reactive UV sites, the number of sites analyzed average 12 for the 9 Å value and 11 for the 8 Å value in the three structures. For the potential UVA-s^4^U sites maximum internucleotide distances of 16 Å and 14 Å limits the number of potential nucleotide pairs to an average of 510 for the 16 Å cut-off value and 305 for the 14 Å cut-off value in the three structures. For the reactive UVA-s^4^U sites, the number of sites analyzed in the three structures average 14 for the 9 Å value and 11 for the 8 Å value, respectively [see Additional file 2].

Comparisons of the frequencies of A minor motif interactions, Mg^2+^-mediated bridges and Mg^2+ ^interactions were determined for 8 Å cut-off values for the UV sites and 14 Å cut-off values for UVA-s^4^U sites. Similar differences are seen between reactive and unreactive nucleotide pairs at these shorter cut-off distance values for both types of photocrosslinking sites for the frequencies of A minor motif interactions, Mg^2+^-mediated bridges, and Mg^2+ ^binding [see Additional file 2].

Comparisons of B factors, hydrogen bonding values, and packing values were determined for 9 Å and 8 Å cut-off values for the UV sites and for 16 Å and 14 Å cut-off values for the UVA-s^4^U sites. There are similar differences in the B factors for the reactive and unreactive sites at different cut-off values [see Additional file 2]. In the comparison of hydrogen bonding differences between reactive and unreactive UV sites, there are similar differences for data selected at 9 Å, and smaller but still significant differences for data selected at 8 Å. For the reactive and unreactive potential s^4^U sites the differences in the hydrogen bonding levels are similar at all cut-off distance values [see Additional file 2]. For the atom count comparison, the differences between the values for the reactive and unreactive UVA-s^4^U sites are similar and statistically significant at all cut-off distance values and there is no trend in these differences as a function of the cut-off distance value. For the UV sites, the differences between the reactive and unreactive sites are smaller and are not quite significant for the 8 Å cut-off distance. The trend in the decreasing difference is due to the general decrease in the neighboring atom count values for both the reactive and unreactive sites as nucleotide pairs at smaller internucleotide distances are measured [see Additional file 2]. However, when average atom count values are divided into groups according to their internucleotide distances there were smaller values for the atom count for reactive nucleotide pairs compared to the unreactive potential nucleotide pairs in a large majority of the intervals [see Additional file 2]. This includes seven of seven internucleotide distance intervals for the UVA-s^4^U data and five of seven internucleotide distance intervals for the UV data.

## Discussion

The comparisons presented here address the underlying structural reasons for high flexibility within the 16S rRNA at some sites in the 30S ribosomal subunit structure and the lack of similar flexibility at many other sites. The comparisons exploit the dependence of photoreactivity on conformational flexibility [26]. Of the several general structural features and interactions that might affect the flexibility, our data indicate that the two that are seen at statistically significantly different levels in the reactive flexible nucleotide pairs compared to the unreactive inflexible pairs are hydrogen bonding and the number of close-by neighboring nucleotides. These both occur at higher levels at the inflexible unreactive sites.

The observation that there are larger average packing densities associated with the nucleotide pairs that have lower flexibilities has two consequences in 16S rRNA. First, there are extensive regions in the 16S rRNA tertiary structure where the biochemical data match the crystal structure [23]. The data here indicate that these regions overall must have high packing density and, in fact, have packing values close to the values seen in double-stranded helical regions. This result indicates that the arrangements of the single-stranded residues involve a high frequency of instances where there are stacking interactions on both sides of the nucleobase surface. Second, for the nucleotide pairs that are reactive in photocrosslinking, usually there are nucleotide pairs in their vicinity that are identified as potential crosslinking sites, but have higher values for neighbor packing or for hydrogen bonding. This arrangement suggests that there may be much more movement between two of the nucleotides and less for other pairs of nucleotides in this type of region. This would result in specific and directional flexibility rather than a general flexibility.

The nature of the underlying organization of RNA tertiary structure recently was addressed by Laederach *et al*. [39] who investigated the relative orientation of the nucleobase planes in RNA structures; the method they developed was applied to 331 structures available in the RNA data base. The majority of the structures contain the RNA bases in coaxial arrangements that indicate extensive positioning of bases all in the same direction in the structure. In the second most common arrangement, the RNA bases are all in the same plane, an arrangement which will also result in preferred base-base interactions. Laederach *et al*. interpreted the overall properties of the RNAs to be a consequence of the base stacking propensity in non-helical as well as in helical regions resulting in favorable stable structures due the maximization of hydrophobic interactions and is compatible with very compact structures due to the high regularity in base stacking. The rRNAs were also included in their analysis and they were notable because they showed a random distribution of base orientations [39]. However, the rRNAs are by far the largest structures analyzed and both the large and small rRNAs are composed of multiple secondary and tertiary structure domains, possibly allowing independence in the behavior of different regions.

We also observed differences in the levels of hydrogen bonding in the 16S rRNA in reactive compared to unreactive sites that accounts further for flexibility differences. Hydrogen bonding has not usually been investigated explicitly in proteins as a factor related to structural stability due to the predominance of hydrophobic interactions in determining folding energy [3,40,41]. However, for nucleic acids that have greater capacity of hydrogen bonding this may play a larger role in establishing the inflexibility or flexibility. The larger extent of hydrogen bonding we observed in the unreactive nucleotide pairs is not a direct consequence of high packing density because there is a modest correlation between neighbor them. Therefore, changes in hydrogen bonding potentially could act independently of changes in packing density in determining the local flexibility.

Our data can be compared to the conclusions recently reported by Fulle and Gohlke [42] who investigated improvements in computational methods for predicting flexibility in RNA. In their analysis, the identification of both van der Waals interactions and hydrogen bonding interactions were important in determining the degrees of freedom of motion for each nucleotide unit. This allowed calculation of a flexibility index that was correlated well to crystallographic B factors and also allowed calculation of molecular motions using constrained geometrical simulations that were well correlated to NMR measurements [42]. Our analysis and the Fulle and Gohlke analysis used very different experimental data and approaches, but both conclusions point to the importance of hydrogen bonding and non-covalent contacts as critical in differentiating flexible and inflexible sites.

It is difficult to understand the apparent absence of differences between reactive and unreactive sites for the other factors with regards to RNA flexibility. The extent of protein contacts, interactions with Mg^2+^, the presence of Mg^2+^-mediated bridges between strands and the presence of the A minor motif interaction do not show significant differences between the reactive and unreactive nucleotide pairs. This is surprising given that there is ample evidence for all of these factors in stabilizing the RNA tertiary structures. In the 16S rRNA, protein-RNA interactions and Mg^2+^-mediated bridge interactions are found at the some of the photoreactive sites, and with regard to promoting or inhibiting photocrosslinking they probably have mixed consequences. These factors help to organize RNA segments from distant parts of the secondary structure and this would promote the formation of sites where there could be interacting nucleotides. On the other hand, if RNA protein interactions or Mg^2+^-mediated bridges induce specific stable structures, the flexibility at those sites could be reduced. In this regard, A-minor interactions in the rRNAs act as scaffolding in the three dimensional structure and act to stabilize the structure [31]. The prospect that there might be conformational switches at some sites where there is conditional formation of the A-minor motif-mediated interactions has been suggested [31]. In any regard, the overall frequencies of the Mg^2+^-mediated bridges and A-minor motif interactions are low and these interactions could only be involved in a small fraction of all sites that are inflexible in the tertiary structure.

## Conclusion

In this study, we evaluated features of the 16S rRNA structure involved in determining the conformational flexibility at the level of internucleotide movements. This approach was possible because we could identify a class of nucleotide pairs based on their internucleotide geometry that were excellent candidates for photocrosslinking, but were not reactive due to lack of flexibility. The extent of hydrogen bonding and packing density both are higher in the nucleotide pairs that are photochemically unreactive, but other features including interactions with proteins and with Mg^2+ ^are similar between the two classes of sites. This non-uniform distribution of hydrogen bonding and packing density in the RNA is unexpected and should be connected to intrinsic ribosome motions.

The differences between the flexible and inflexible nucleotide pairs do not prove causality between hydrogen bonding, molecular packing and conformational inflexibility. However, intuitively this is a likely hypothesis and is supported by the studies in proteins that independently addressed this problem and uncovered the connection between local packing density and molecular flexibility.

## Methods

### Crosslinking data and structure measurements

UV-induced photocrosslinking sites in the *E. coli *16S rRNA and their frequencies resulting from irradiation of 30S ribosomes with a single excimer laser pulse are from Shapkina *et al*. [28]. The UVA – s^4^U-induced crosslinking sites and their frequencies determined after 10 min irradiation of ribosomes containing internally substituted s^4^U are from Nanda and Wollenzien [25].

Distances and angles between nucleotide pairs in the 16S rRNA were calculated from the atomic coordinates of the *T. thermophilus *30S ribosome structure (PDB ID 1FJF) from Wimberly *et al*. [11] and from the two 30S ribosome structures (PDB ID 2AVY and 2AWY) of Shuwirth *et al*. [13]. The sequence numbering in *T. thermophilus *compared to *E. coli *16S rRNAs was according to Brodersen *et al*. [29]. For both UV-induced and UVA-s^4^U-induced photocrosslinking, the reactions require alignment and direct contact of the reactive bonds, including co-planarity of the two nucleobase planes. The photocrosslinks made by irradiation with UV light involve the double bonds of C5-C6 atoms of pyrimidines or the double bonds at the N7-C8 atoms of purines to form cyclobutane or cycloazotine adducts [43-45]. For the UVA-induced crosslinking in s^4^U-containing RNA, the C4-S4 double bond of the s^4^U and the double bond at the C5-C6 atoms of pyrimidines or the double bond at the N7-C8 atoms of purines are initially involved, and these subsequently undergo elimination reactions to result in C4-C6 or C4-C8 internucleotide bonds [46-48]. Positions of the midpoints of photoreactive bonds were calculated from atom coordinates – C5/C6 for pyrimidines and N7/C8 for purines involved in UVB-induced crosslinking, C4/O4 for the nucleotide identified as an s^4^U in the s^4^U substituted 30S subunits, and C5/C6 for pyrimidines or N7/C8 for purines that are the partners for s^4^U (see Figure 2). Torsion angles between base planes were calculated by first determining the planes of each base using the coordinates for the C2, C4 and C6 atoms, followed by the calculation of the torsion angle between vectors normal to the planes. The angles between reactive bonds were calculated from vectors through the C5-C6 atoms of pyrimidines, through C4-O4 for s^4^U or through the N7-C8 atoms of purines, and were calculated by the standard expression for the angle between vectors in three dimensional space.

Nucleotide pairs for potential UV-photocrosslinking were considered if the distance between the bonds that should be photoreactive was 10.5 Å or less. This cut-off distance includes an average of 14 of the observed UV crosslinking sites in each of the structures and excludes the remainder that have exceptionally long internucleotide distances. Nucleotide pairs separated by less than 40 nucleotides in the primary sequence were excluded because those interactions would not be detected by the experimental methods. Nucleotides pairs that were within a regular double-stranded region were also excluded because crosslinking within base-paired regions is not expected [49]. Randomly selected nucleotide pairs were visually inspected and several were found to be close by virtue of side-by-side arrangements in which the nucleobases were nearly co-planar. This arrangement was not seen in the crosslinked nucleotide pairs, so these pairs were computationally identified and removed. To do this, for each nucleotide pair, the closest distance between the first nucleotide and the plane of the nucleobase of the second was calculated and *vise versa*. Nucleotide pairs that had both of these values at less than 1.2 Å were excluded from the lists.

For the potential UVA-s^4^U photocrosslinks, the initial list consisted of nucleotide pairs in which there was a partner nucleotide for each s^4^U within 18 Å calculated between photoreactive bonds. Eighteen Å is the distance that includes an average of 17 of the reactive observed UVA-s^4^U photocrosslinks in the three structures. Nucleotide pairs separated by less than 40 nucleotides, within a regular double-stranded region, or in side-by side arrangements were excluded from the list using the methods described above.

### Counting of A minor motif, Mg^2+ ^and protein interactions

A list of A minor motif contacts was created from Noller [31] based on the geometry of the type I and type II interactions [30] and the *E. coli *II structure [13]. The lists of the nucleotide pairs at the reactive observed photocrosslinking sites and at the unreactive potential sites were manually checked against the A minor motif interaction list to determine frequencies. Similarly lists of nucleotide pairs that share contacts with the same Mg^2+ ^ion were generated from the data of the two *T. thermophilus *30S structures (PDB ID 2j00 and 2j01) reported by Selmer *et al*. [34]. Lists of nucleotides that are in proximity to Mg^2+ ^ions were also generated from the Selmer *et al*. data. The lists of the reactive observed nucleotide pairs and the unreactive potential nucleotide pairs were manually checked against the Mg^2+ ^list to determine the number of instances where Mg^2+ ^bridges occurred or the frequency of Mg^2+ ^binding. A Mg^2+ ^ion in a ± 2 nt window were considered an interaction.

### Identification of tertiary hydrogen bonds

Hydrogen bonding is known to be highly directional [36,37] so a method was developed to identify hydrogen bonds in which the donor and acceptor participants were pointed at each other in the correct way as well as being separated by the correct distance. To do this we used the pyrimidine and purine structures to find the expected directions of hydrogen bonding. For planar nucleobase structures with approximately 120° bond angles, the direction of hydrogen bonding can be estimated by the direction of bonds within the nucleobase ring structure [36]. For instance, in cytidine the two potential hydrogen bonds at N4 are in the direction of the C5-C4 or N3-C4 bonds, and the direction of the hydrogen bond at N3 is in the direction of C5-C4 bond. Cut-off values that allow dispersion in the distances and angles between potential hydrogen bonding heavy atoms were adjusted so that the correct number of hydrogen bonds were identified in the double stranded regions of the 30S subunit. These cut off values then allowed identification of additional appropriate hydrogen bonding interactions in the tertiary structure. Potential hydrogen bonding sites were identified if the vectors between the donor and acceptor atoms were coincident and pointing at each other and were at the correct distance. For hydrogen bonding at the 2' OH or at the phosphate oxygens, a distance criterion, 2.8 to 3.1 Å between donor and acceptor was used.

Hydrogen bonds were also determined and confirmed with the program HBexplore (version 2.0.1, ref [38]), which gave a similar results. The total number of hydrogen bonds for each nucleotide of a nucleotide pair was listed on an Excel spread sheet, which was used for calculations.

### Calculation of neighbor packing density around the nucleotides

A counting method was used to calculate the packing density [7,50]. The counts did not include the nucleotide unit itself or the nucleotide that is the observed or potential crosslinking partner to ensure that only possible interfering nucleotides would contribute to the values. The number of heavy atoms within a six Å radius of each nucleotide was determined using the midpoint between the C3 and N6 atoms of pyrimidines or from the midpoint between the N1, C2 and C8 of purines as the center of measurement. Neighbor count values were listed separately for each nucleotide in each nucleotide pair and the lesser value of the two was listed.

### Statistical Analysis

Correlations between pairs of measurements were calculated using r, the Pearson product-moment correlation coefficient. Linear regression and multiple linear regression analysis to relate the crosslinking frequencies and geometry factors were done using R software . The models for the linear regression analyses were chosen to include the parameters that have the largest correlations to the frequencies. The form of the second non-linear regression equation, involving a product of reciprocal internucleotide distance and sine or cosine of internucleotide angle between base planes, was used to avoid negative beta values, which are physically unreasonable. The z test [51] was used to compare the properties with continuous values of the reactive and unreactive nucleotide pairs. The hypothesis that the average values of the two populations were the same was tested using p value [see ].

#### Illustrations

The atomic coordinates for the *E. coli *II 30S subunit structure [13] were used for figures 1 and 2. The figures were prepared with the program Ribbons [52].

## Abbreviations

BPA: (nucleo)base plane angle; RBD: reactive bond distance; s^4^U: 4-thiouridine; RMSD: root mean square difference; Y: pyrimidine nucleotide; R: purine nucleotide.

## Authors' contributions

WH designed and carried out calculations of the structural parameters and wrote the manuscript. SJG performed statistical calculations. PW wrote the manuscript. All authors read and approved the final manuscript.

## Supplementary Material

Additional file 1 **Comparison of the properties of photoreactive and unreactive nucleotide pairs in the 16S rRNA by additional criteria**. The file contains analyses that compare reactive and unreactive nucleotide pairs by additional geometrical and stereochemical criteria. Click here for file

Additional file 2 **Comparison of the properties of photoreactive and unreactive nucleotide pairs in the 16S rRNA after selection by different internucleotide distances**. The file contains analyses that compare properties of the reactive and unreactive nucleotide pairs after selection of the data by the criteria of different internucleotide distances. Click here for file
