# Supplementary material for: Hydrogen bonding and packing density are factors most strongly connected to limiting sites of high flexibility in the 16S rRNA in the 30S ribosome
Source: BMC Struct Biol. 2009 Jul 30;9:49. doi: 10.1186/1472-6807-9-49 (PMC2731775; doi:10.1186/1472-6807-9-49)
Supplement: Additional file 1 — Comparison of the properties of photoreactive and unreactive nucleotide pairs in the 16S rRNA by additional criteria. The file contains analyses that compare reactive and unreactive nucleotide pairs by additional geometrical and stereochemical criteria. [file 1472-6807-9-49-S1.pdf]

Additional file 1: Comparison of the properties of photoreactive and unreactive nucleotide pairs in the 16S rRNA by additional criteria.

Additional Table 1. Correlation coefficients between UVB and UVA-s^4U crosslinking frequencies and internucleotide geometry measures. Geometry features and B factors in three different 30S structures were calculated and used to determine correlation coefficients to determine which geometry features consistently show correlation to the crosslinking frequencies.

Additional Figure 1. Spatial arrangement of reactive and unreactive UV sites. The arrangements of the nucleobase parts of nucleotide pairs from the sites where there is observed UV-induced photocrosslinking and from sites that were identified by regression analysis to have suitable geometry were examined to see if there are differences in the arrangements of the reactive and inflexible unreactive sites.

Additional Figure 2. Spatial arrangement of reactive and unreactive s^4U sites. The arrangements of the nucleobase parts of nucleotide pairs from the sites where there is observed UVA-s^4U-induced photocrosslinking and from sites that were identified by regression analysis to have suitable geometry were examined to see if there are differences in the arrangements of the reactive and inflexible unreactive sites.

Additional Table 2. Compositional and stereochemical properties of reactive and unreactive sites. Properties of the nucleotide pairs at the reactive and unreactive sites are summarized. The properties include features of the internucleotide arrangement that were quantified from the visual inspection of the nucleotide pairs. Properties were compared using the *E. coli* II and *T. thermophilus* structures.

Additional Table 3. Neighbor atom count for reactive and unreactive nucleotide pairs in the three 30S structures. Neighbor atom count is summarized for reactive and non-reactive nucleotide pairs evaluated from each of the three ribosome structures.

Additional Table 4. Comparison of Expected and Measured Values of Hydrogen Bonding and Neighbor Atom Count. Expected values of properties for the reactive and unreactive nucleotide pairs were calculated based on the composition of nucleotides in single-stranded and double-stranded regions. These are compared to the values measured in the structures. The *E .coli* II structure was used for all measurements.

Additional Table 1. Correlation coefficients between UV and UVA-s^4U crosslinking frequencies and internucleotide geometry measures in different 30S structures

		**Correlation coefficients**[1]			
**Reaction**	**Measure**[2]	***E. coli* I**	***E. coli* II**	***T. thermophilus***	**Ave.**
**UV**	Freq. vs 1/(C1'-C1')	0.627	0.495	0.389	0.504
	〃 vs 1/(RBD)	- 0.004	- 0.035	0.110	0.024
	〃 vs 1/(RBA	0.165	- 0.080	0.693	0.259
	〃 vs 1/(BPA)	- 0.181	- 0.003	0.230	0.015
	〃 vs B factor	0.464	0.402	0.296	0.386
**UVA-s^4U**	Freq. vs 1/(C1'-C1')	0.388	0.389	0.538	0.438
	〃 vs 1/(RBD)	0.414	0.348	0.351	0.371
	〃 vs 1/(RBA	- 0.130	- 0.128	- 0.189	- 0.149
	〃 vs 1/(BPA)	0.066	- 0.099	0.252	0.073
	〃 vs B factor	0.567	0.317	0.269	0.384
**UV**	BPA vs RBD	0.410	0.447	0.555	0.471
**UVA-s^4U**	BPA vs RBD	0.394	0.366	0.783	0.514

[1]The Pearson product moment correlation coefficients are $(\Sigma (x_i - <x>)(y_i - <y>))/ ((n-1) s_x s_y)$ where $<x>$ and $<y>$ are the sample means and s_x and s_y are the sample standard deviations of x and y. The geometry and B factor data are calculated from the 30S subunit parts of the *E. coli* I and *E. coli* II 70S structures and the *T. thermophilus* 30S structure.

[2]Measures are: C1'- C1', distance between C1' atoms; RBD, distance between centers of reactive bonds; RBA, angle between the reactive bonds; BPA, angle between the planes of the bases. The B factor is the average calculated for the heavy atoms of the nucleobases in each nucleotide pair.

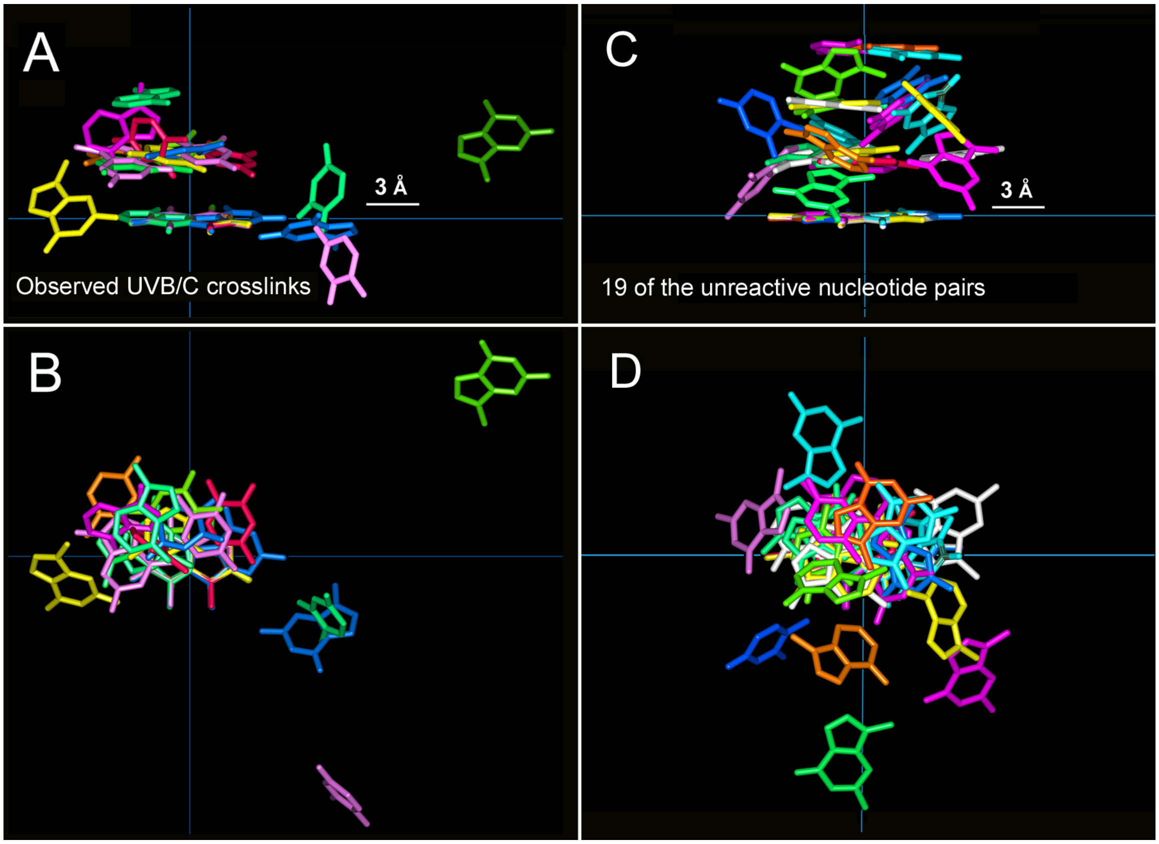

Additional Figure 1. Three dimensional arrangements of nucleobases of the nucleotide pairs at observed and at potential UV photocrosslinking sites. A and B. Perpendicular views of the nucleobases from the observed UV crosslinks. The photoreactive bond of one of the nucleobases of each pair was picked and superimposed with a reference nucleobase so that all of the partners occupy the same hemisphere. The crosshairs intersect at the photoreactive bond. A. View of the arrangements in a direction nearly parallel to the plane of the reference nucleobase. B. View of the arrangements in a direction perpendicular to the plane of the reference nucleobase. C and D. Perpendicular views of nucleobases from potential UV crosslinked nucleotide pairs. Thirty one pairs were picked for inspection; these have distances between reactive bonds, or distances between C1'- C1' atoms, that matched with those seen in the observed pairs, but otherwise were picked randomly from the whole list of 674 potential pairs. Nucleobase pairs were arranged in the same way as for the observed pairs. The views are the same as in panels A and B.

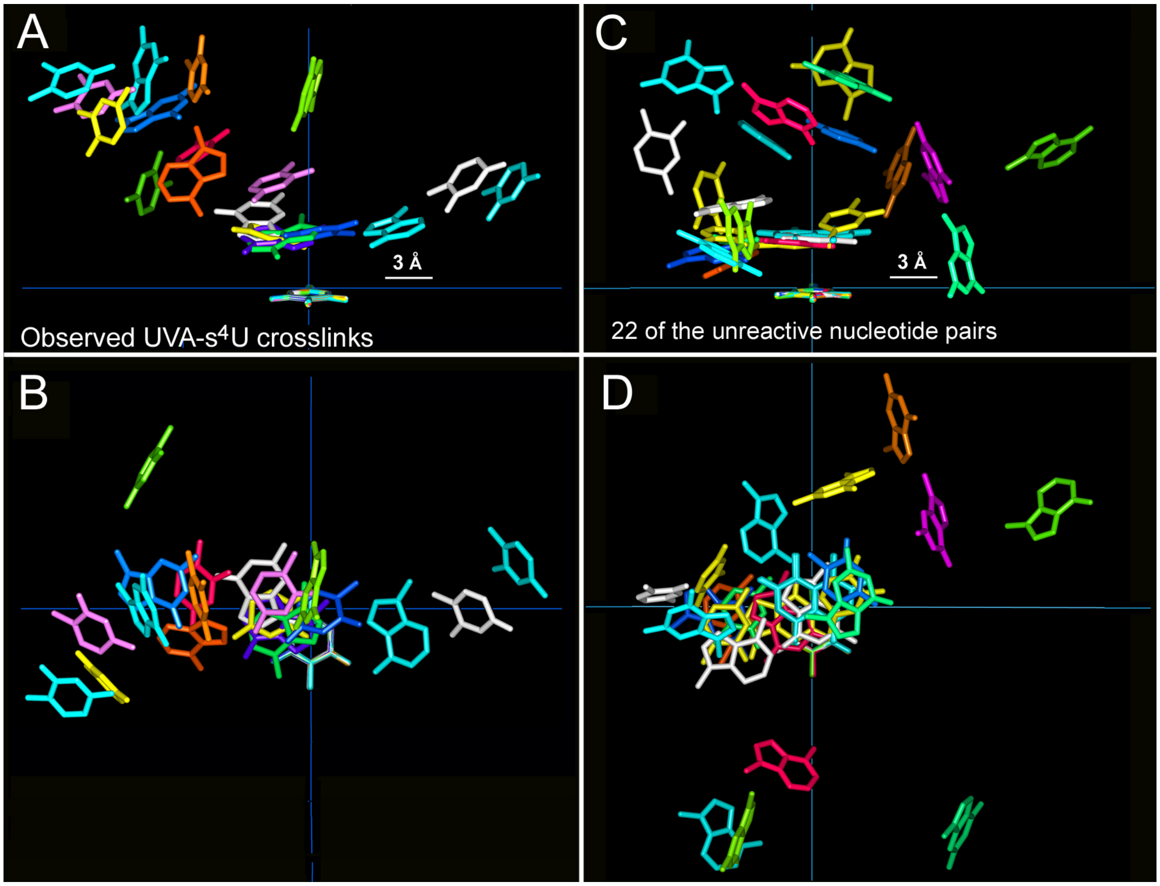

Additional Figure 2. Three dimensional arrangements of nucleobases of the nucleotide pairs at observed and at potential UVA-s^{4}U photocrosslinking sites. A and B. Perpendicular views of the nucleobases of the observed UVA-s^{4}U crosslinks. The photoreactive bond of the nucleobase that must be the s^{4}U of each pair was picked and superposed with one (of two possible orientations) of the reference s^{4}U so that all of the partners occupy the same hemisphere. The crosshairs intersect at the photoreactive bond. A. View of the arrangements in a direction nearly parallel to the plane of the reference nucleobase. B. View of the arrangements in a direction perpendicular to the plane of the reference nucleobase. C and D. Perpendicular views of nucleobases from potentially UVA-s^{4}U crosslinked nucleotide pairs. Nucleobase pairs were arranged in the same way as for the observed pairs. Thirty two pairs were picked for inspection; these have distances between reactive bonds, or distances between C1'- C1' atoms that match those seen in the observed pairs, but otherwise were picked randomly from the whole list of 928 potential pairs.

Additional Table 2. Comparison of properties of nucleotide pairs using *T. thermophilus* and *E. coli* II structures

T. thermophilus

	Composition[1]			Orientation[2]		Base overlap[3]		Distance Differences[4]		
**UV nt. Pairs**	**Y/Y**	**Y/R**	**R/R**	**Anti**	**Syn**	**Cis**	**Trans**	**RBD**	**C1'-C1'**	**RMSD**
Reactive pairs (16)	8	6	2	13	3	10	6	8.0 ± 4.4	9.4 ± 3.8	2.7 ± 2.0
Unreactive pairs (19)	4	9	6	11	8	13	6	7.3 ± 2.0	8.4 ± 1.5	1.8 ± 1.0
**UVA-s^{4}U nt. pairs**	**s^{4}U/Y**	**s^{4}U /R**		**Anti**	**Syn**	**Cis**	**Trans**	**RBD**	**C1'-C1'**	**RMSD**
Reactive pairs (18)	11	8		19	0	13	6	11.0 ± 5.5	13.2 ± 4.9	4.6 ± 2.3
Unreactive pairs (22)	12	10		14	8	12	10	9.7 ± 3.6	11.5 ± 2.9	3.5 ± 1.6

***E. coli* II**

	Composition[1]			Orientation[2]		Base overlap[3]		Distance Differences[4]		
**UV nt. Pairs**	**Y/Y**	**Y/R**	**R/R**	**Anti**	**Syn**	**Cis**	**Trans**	**RBD**	**C1'-C1'**	**RMSD**
Reactive pairs (16)	9	6	3	13	3	11	6	8.5 ± 4.5	9.4 ± 4.4	2.6 ± 2.6
Unreactive pairs (19)	4	16	11	20	111	13	18	8.4 ± 2.1	9.2 ± 3.0	2.4 ± 0.9
**UVA-s^{4}U nt. pairs**	**s^{4}U/Y**	**s^{4}U /R**		**Anti**	**Syn**	**Cis**	**Trans**	**RBD**	**C1'-C1'**	**RMSD**
Reactive pairs (18)	12	9		19	0	13	6	11.5 ± 5.6	13.1 ± 5.2	4.1 ± 2.4
Unreactive pairs (22)	6	26		20	12	19	13	11.1 ± 3.9	13.1 ± 5.1	3.9 ± 1.7

[1]Pyrimidines (Y) or purines (R) are indicated.

[2]Orientation refers to the directions of the glycosidic bonds in the two nucleotides away (anti) or towards (syn) each other.

[3] Base overlap refers to the arrangement of the nucleobases after best alignment to the model structures; arrangements in which the bases are stacked over each other are cis, arrangements in which bases are not stacked are trans.

[4]Distances are between the expected photoreactive bonds of the pair (RBD) and between C1'- C1' atoms of the nucleotides in the pair (C1'). The root mean square deviation (RMSD) is calculated from the geometry observed in the tertiary structure compared to the geometry needed for photoreaction. Only the intra-cyclic heavy atoms of the nucleobases were included in the RMSD calculation.

Additional Table 3. Comparison of neighbor atom count for reactive and unreactive nucleotide pairs using different atomic structures

UV-induced photocrosslinking sites – both nt.

Nucleotide pair	Number of nt. pairs			Ave. atom count around both nt.[1]					
	**T. t.**	**E.c. I**	**E.c. II**	**T. t.**	**E.c. I**	**E.c. II**	**Ave. ± S.D.**	**n**	**z value**
Reactive	14	13	15	23.5 ±10.3	21.4 ±11.4	21.2 ± 9.2	22.0 ±10.3	42	
Unreactive	714	768	674	28.5 ± 9.6	27.6 ± 9.3	27.9 ± 9.0	28.0 ± 9.3	2156	- 4.32
z values for differences between values in each structure				- 1.93	- 2.63	- 2.94			

UV-induced photocrosslinking sites – lower-packed nt.

Nucleotide pair	Number of nt. pairs			Ave. atom count around lower-packed nt.[1]					
	**T. t.**	**E.c. I**	**E.c. II**	**T. t.**	**E.c. I**	**E.c. II**	**Ave. ± S.D.**	**n**	**z value**
Reactive	14	13	15	14.3 ±10.3	15.7 ±11.2	14.7 ±11.5	14.9 ±11.0	42	
Unreactive	714	768	674	24.0 ± 9.8	22.4 ± 9.4	23.2 ± 9.1	23.2 ± 9.4	2156	- 5.93
z values for differences between values in each structure				- 3.66	- 2.82	- 3.68			

UVA-s^4U-induced photocrosslinking sites – both nt.

Nucleotide pair	Number of nt. pairs			Ave. atom count around both nt.[1]					
	**T. t.**	**E.c. I**	**E.c. II**	**T. t.**	**E.c. I**	**E.c. II**	**Ave. ± S.D.**	**n**	**z value**
Reactive	18	16	16	27.7 ± 9.9	24.3 ±11.6	24.8 ±12.8	25.6 ±11.4	50	
Unreactive	940	893	928	31.4 ± 7.8	30.5 ± 8.8	30.4 ± 8.7	30.8 ± 8.4	2761	- 4.50
z values for differences between values in each structure				- 1.98	- 2.94	- 2.70			

UVA-s^4U-induced photocrosslinking sites – lower-packed nt.

Nucleotide pair	Number of s^4U			Ave. atom count around s^4U					
	**T. t.**	**E.c. I**	**E.c. II**	**T. t.**	**E.c. I**	**E.c. II**	**Ave. ± S.D.**	**n**	**z value**
Reactive	8	8	8	17.4 ±11.0	18.9 ±13.0	17.9 ±14.4	18.1 ±12.8	24	
Unreactive	105	105	105	32.3 ± 7.0	29.0 ± 9.5	28.8 ± 9.4	30.0 ± 8.6	315	- 5.93
z values for differences between values in each structure				- 5.56	- 2.82	- 3.05			

[1]The average values and standard deviations for reactive and unreactive nucleotide pairs in each structure are indicated.

[2] The z-value is the difference in the averages in units of weighted standard error of the mean (51). The null hypothesis, that the populations have the same averages, can be rejected at the 5% and 1% level of significance if $|z| \geq 1.96$ and $|z| \geq 2.56$, respectively.

Additional Table 4. Comparison of Expected and Measured Hydrogen Bonding and Neighbor Atom Count

H bonds at UV sites	Fraction ss-ds	H bonds expected	H bonds measured
Reactive sites	0.21	0.81 ± 1.14	1.12 ± 0.91
Unreactive sites	0.38	0.98 ± 1.25	1.70 ± 0.85

Average H bonds at all single-stranded nucleotides: 0.598 ± 0.916 (n = 675); H bonds at double-stranded nucleotides: 2.60 ± 0.56 (n = 130)

H bonds at UVA-s^4U sites	Fraction ss-ds	H bonds expected	H bonds measured
Reactive sites	0.22	0.73 ± 1.09	1.05 ± 0.69
Unreactive sites	0.54	1.05 ± 1.35	1.76 ± 0.77

Average H bonds at single-stranded s^4U: 0.43 ± 0.76 (n = 73); H bonds at all single-stranded nucleotides: 0.598 ± 0.916 (n = 675); H bonds at double-stranded nucleotides: 2.60 ± 0.56 (n = 130)

Neigh. Atom count UV sites	Fraction ss-ds	Neigh. atom cnt. expected	Neigh. Atom cnt. measured
Reactive sites	0.21	24.2 ± 9.4	22.0 ± 10.2
Unreactive sites	0.38	24.6 ± 8.8	28.0 ± 9.3

Average neighbor atom count around single-stranded nucleotides: 23.7 ± 9.9; around double-stranded nucleotides: 28.7 ± 3.9

Neigh. Atom count UVA-s^4U sites	Fraction ss-ds	Neigh. Atom cnt. expected	Neigh. Atom cnt. measured
Reactive sites	0.22	23.6 ± 9.5	25.6 ± 11.4
Unreactive sites	0.54	24.4 ± 8.7	30.8 ± 8.4

Average neighbor atom count around single-stranded s^4U: 22.4 ± 10.2; around single-stranded nucleotides: 23.7 ± 9.9; around double-stranded nucleotides: 28.7 ± 3.9.
