# Supplementary material for: Hydrogen bonding and packing density are factors most strongly connected to limiting sites of high flexibility in the 16S rRNA in the 30S ribosome
Source: BMC Struct Biol. 2009 Jul 30;9:49. doi: 10.1186/1472-6807-9-49 (PMC2731775; doi:10.1186/1472-6807-9-49)
Supplement: Additional file 2 — Comparison of the properties of photoreactive and unreactive nucleotide pairs in the 16S rRNA after selection by different internucleotide distances. The file contains analyses that compare properties of the reactive and unreactive nucleotide pairs after selection of the data by the criteria of different internucleotide distances. [file 1472-6807-9-49-S2.pdf]

**Additional file 2: Comparison of the properties of photoreactive and unreactive nucleotide pairs in the 16S rRNA after selection by internucleotide distance.**

**Additional Table 5.** Comparison of A minor motif interactions, $Mg^{2+}$ bridges and $Mg^{2+}$ at nucleotide pairs in reactive observed nucleotide pairs and unreactive potential nucleotide pairs for pairs within 8 Å and 14 Å.

**Additional Table 6.** Comparison of B factors, hydrogen bonding and neighbor atom count around reactive and unreactive nucleotide pairs for pairs within 9 Å and 16Å.

**Additional Table 7.** Comparison of B factors, hydrogen bonding and neighbor atom count around reactive and unreactive nucleotide pairs for pairs within 8 Å and 14Å.

**Additional Figure 3.** Plots of z values for differences in the properties of reactive and unreactive nucleotide pairs for data selected with successive internucleotide distance cut-off values.

**Additional Table 8.** Comparison of atom count around reactive and unreactive nucleotide pairs for different internucleotide distance intervals.

**Additional Table 5. Comparison of frequency of A minor motif interactions and $Mg^{2+}$ binding in the vicinity of the reactive and unreactive nucleotide pairs for pairs at < 8 Å and < 14 Å[1]**

| **UV reactions** | Nucleotide Pairs | | Statistics[2] | |
|---|---|---|---|---|
| Parameter | Reactive | Unreactive | z value | p value |
| Number of nt. pairs in A minor motif[3] | 0 of 12 nt. pairs | 4 of 355 nt. pairs | -0.179 | 0.869 |
| $Mg^{2+}$ in vicinity of nt.[4] | 46 $Mg^{2+}$ at 48 nt. | 1028 $Mg^{2+}$ at 1420 nt. | 0.675 | 0.525 |
| $Mg^{2+}$ bridges in vicinity of nt. pair[5] | 2 of 24 nt. pairs | 36 of 710 nt. pairs | 0.803 | 0.457 |

| **UVA-$s^4$U reactions** | Nucleotide Pairs | | Statistics | |
|---|---|---|---|---|
| Parameter | Reactive | Unreactive | z value | p value |
| Number of nt. pairs in A minor motif[6] | 0 of 13 nt. pairs | 8 of 776 nt. pairs | -0.147 | 0.888 |
| $Mg^{2+}$ in vicinity of nt.[7] | 23 $Mg^{2+}$ at 42 nt. | 1544 $Mg^{2+}$ at 1930 nt. | -1.159 | 0.291 |
| $Mg^{2+}$ bridges in vicinity of nt. pair[8] | 0 of 26 nt. pairs | 17 of 1552 nt. pairs | -0.159 | 0.879 |

[1] Observed reactive and potential unreactive nucleotide pairs were considered if distances between nucleotides (at photoreactive bonds) was less than 8 or 14 Å for the UV or UVA-$s^4$U sites, respectively.

[2] The significance of the z values and p values are the same as described in the foot notes to Table 3.

[3] Twelve nucleotide pairs in reactive observed sites and 102, 107 and 146 nucleotide pairs at unreactive potential sites in the *E. coli* I and II [13] and *T. thermophilus* structures [11] were evaluated using the list of A minor motif interactions [31].

[4] Twenty four nucleotides from the reactive observed UV sites and 710 nucleotides from the unreactive nucleotide pairs in the *E. coli* I and II [13] and *T. thermophilus* structures [11] were evaluated using the lists of $Mg^{2+}$ interaction in the *T. thermophilus* I and II structures [34].

[5] Twelve nucleotide pairs in observed crosslinks and 355 nucleotide pairs at unreactive sites in the *T. thermophilus* [11] and *E. coli* I and II [13] structures were evaluated using the lists of the $Mg^{2+}$ interactions sites in the *T. thermophilus* I and II structures [34].

[6] Thirteen nucleotide pairs in observed crosslinks and 211, 217 and 348 nucleotide pairs at unreactive sites in the *E. coli* I and II [13] and *T. thermophilus* [11] structures were evaluated using the list of A minor motif interactions [31].

[7] Twenty one nucleotides from the observed reactive UVA-$s^4$U sites and nucleotides in the unreactive nucleotide pairs in the and *E. coli* I and II [13] and *T. thermophilus* [11] structures were evaluated using the lists of $Mg^{2+}$ interaction in the *T. thermophilus* I and II structures [34]. Each $s^4$U in observed or potential sites was evaluated only once.

[8] Thirteen nucleotide pairs in observed crosslinks and 776 nucleotide pairs at unreactive sites in the *T. thermophilus* [11] and *E. coli* I and II [13] structures were evaluated using the lists of the $Mg^{2+}$ interactions sites in the *T. thermophilus* I and II structures [34].

**Additional Table 6. Comparison of B factors, hydrogen bonding and neighbor atom count around reactive and unreactive nucleotide pairs for pairs within 9 Å and 16Å**

| **UV reactions** | | Nucleotide Pairs | | | Statistics[3] | |
|---|---|---|---|---|---|---|
| | | Reactive | | Unreactive | | |
| Parameter | n[1] | Ave. ± S.D. | n[2] | Ave. ± S.D. | z value | p value |
| B factor for both nt. | 36 | 64.3 ± 24.1 | 853 | 58.8 ± 24.7 | 1.310 | 0.1907 |
| Value of larger B factor of pair | 36 | 76.9 ± 36.3 | 853 | 67.8 ± 25.5 | 2.056 | 0.0400 |
| H bonds/nt. for both nt. | 36 | 1.15 ± 1.20 | 853 | 1.84 ± 1.05 | -3.839 | 0.0001 |
| H bonds in lesser H-bonded nt. | 36 | 0.51 ± 0.68 | 853 | 1.11 ± 0.93 | -3.827 | 0.0001 |
| Atom count around both nt. | 36 | 21.4 ± 11.6 | 853 | 26.2 ± 9.9 | -2.829 | 0.0050 |
| Atom count – lower-packed nt. | 36 | 15.6 ± 10.5 | 853 | 21.6 ± 9.9 | -3.553 | 0.0004 |

| **UVA-s$^4$U reactions** | | Nucleotide Pairs | | | Statistics | |
|---|---|---|---|---|---|---|
| | | Reactive | | Unreactive | | |
| Parameter | n[4] | Ave. ± S.D. | n[5] | Ave. ± S.D. | z value | p value |
| B factor for both nt. | 46 | 66.9 ± 25.2 | 1529 | 58.7 ± 26.3 | 2.086 | 0.0370 |
| Value of larger B factor of pair | 46 | 75.4 ± 26.8 | 1529 | 68.8 ± 28.6 | 1.545 | 0.1226 |
| Value of s$^4$U B factor | 24 | 72.4 ± 23.6 | 303 | 74.8 ± 32.9 | 0.088 | 0.9302 |
| H bonds/nt. for both nt. | 46 | 1.25 ± 0.97 | 1529 | 1.66 ± 0.78 | -3.486 | 0.0005 |
| H bonds/s$^4$U | 24 | 0.85 ± 0.98 | 303 | 1.38 ± 1.04 | -2.413 | 0.0163 |
| Atom count around both nt. | 46 | 25.5 ± 11.2 | 1529 | 31.1 ± 8.1 | -4.561 | <0.0001 |
| Atom count around the s$^4$U | 24 | 22.7 ± 11.2 | 303 | 29.4 ± 8.8 | -3.514 | 0.0005 |

[1]The number of reactive nucleotide pairs evaluated is 11, 12 and 13 in the *T. thermophilus*, *E. coli* I and *E. coli* II structures respectively, after removal of observed crosslinks that have ≥ 9 Å between reactive bonds and because two crosslinking sites are not present in the *T. thermophilus* structure. The average and standard deviations are weighted averages and standard deviations from three sets of measurements.

[2]The number of measurements of unreactive pairs is 322, 259 and 272 in the *T. thermophilus*, *E. coli* I and *E. coli* II structures respectively, except for the hydrogen bonding frequencies which were evaluated from a representative number of nucleotide pairs in each structure.

[3]z-value is the difference in the averages in units of weighted standard error of the mean. The null hypothesis, that the populations have the same averages, can be rejected at the 5% and 1% level of significance if $|z| \geq 1.96$ and $|z| \geq 2.56$, respectively. p-value is the probability of obtaining the difference in the averages if the null hypothesis is correct, in this case, that the two average values are the same.

[4]The number of reactive nucleotide pairs evaluated is 16, 15 and 15 in the *T. thermophilus*, *E. coli* I and *E. coli* II structures, after removal of observed crosslinks that have ≥16 Å between reactive bonds. For evaluation of H bonds/s$^4$U and atom count around the s$^4$U, there are eight s$^4$Us that are involved in the 18 photocrosslinks that are evaluated in each of the three structures.

[5]The number of measurements of unreactive pairs is 551, 504 and 474 in the *T. thermophilus*, *E. coli* I and *E. coli* II structures respectively, except for the value of the s$^4$U B factor, H bonds/s$^4$U, and atom count around the s$^4$U which were evaluated just once on each of the substituted s$^4$U positions in each structure.

**Additional Table 7 Comparison of B factors, hydrogen bonding and neighbor atom count around reactive and unreactive nucleotide pairs for pairs within 8 Å and 14 Å**

**UV reactions**

| | Nucleotide Pairs | | | | Statistics[3] | |
|---|---|---|---|---|---|---|
| | Reactive | | Unreactive | | | |
| Parameter | n[1] | Ave. ± S.D. | n[2] | Ave. ± S.D. | z value | p value |
| B factor for both nt. | 32 | 66.4 ± 27.7 | 422 | 59.1 ± 23.7 | 1.591 | 0.1122 |
| Value of larger B factor of pair | 32 | 79.4 ± 35.3 | 422 | 68.1 ± 24.7 | 2.410 | 0.0163 |
| H bonds/nt. for both nt. | 32 | 1.14 ± 1.19 | 422 | 1.54 ± 1.07 | -2.022 | 0.0443 |
| H bonds in lesser H-bonded nt. | 32 | 0.46 ± 0.65 | 422 | 0.82 ± 0.98 | -2.040 | 0.0428 |
| Atom count around both nt. | 32 | 20.8 ± 10.8 | 422 | 23.5 ± 9.9 | -1.532 | 0.1261 |
| Atom count – lower-packed nt. | 32 | 15.0 ± 10.2 | 422 | 18.8 ± 9.5 | -2.170 | 0.0306 |

**UVA-$s^4U$ reactions**

| | Nucleotide Pairs | | | | Statistics | |
|---|---|---|---|---|---|---|
| | Reactive | | Unreactive | | | |
| Parameter | n[4] | Ave. ± S.D. | n[5] | Ave. ± S.D. | z value | p value |
| B factor for both nt. | 32 | 68.5 ± 28.8 | 915 | 57.6 ± 25.9 | 2.466 | 0.0144 |
| Value of larger B factor of pair | 32 | 77.3 ± 27.9 | 915 | 62.5 ± 29.8 | 2.930 | 0.0035 |
| Value of $s^4U$ B factor | 24 | 72.4 ± 23.5 | 242 | 68.1 ± 28.0 | 0.727 | 0.4688 |
| H bonds/nt. for both nt. | 32 | 1.11 ± 0.93 | 915 | 1.67 ± 0.79 | -4.142 | <0.0001 |
| H bonds/$s^4U$ | 24 | 0.85 ± 1.24 | 242 | 1.92 ± 1.47 | -3.445 | 0.0007 |
| Atom count around both nt. | 32 | 25.3 ± 11.0 | 915 | 31.2 ± 7.8 | -4.373 | <0.0001 |
| Atom count around the $s^4U$ | 24 | 21.0 ± 11.0 | 242 | 28.0 ± 8.0 | -3.939 | <0.0001 |

[1]The number of reactive nucleotide pairs evaluated is 11, 12 and 9 in the *T. thermophilus*, *E. coli* I and *E. coli* II structures respectively, after removal of observed crosslinks that have ≥ 8 Å between reactive bonds and because two crosslinking sites are not present in the *T. thermophilus* structure. The average and standard deviations are weighted averages and standard deviations from three sets of measurements.

[2]The number of measurements of unreactive pairs is 151, 137 and 134 in the *T. thermophilus*, *E. coli* I and *E. coli* II structures respectively, except for the hydrogen bonding frequencies which were evaluated from a representative number of nucleotide pairs in each structure.

[3]z-value is the difference in the averages in units of weighted standard error of the mean. The null hypothesis, that the populations have the same averages, can be rejected at the 5% and 1% level of significance if $|z| \geq 1.96$ and $|z| \geq 2.56$, respectively. p-value is the probability of obtaining the difference in the averages if the null hypothesis is correct, in this case, that the two average values are the same.

[4]The number of reactive nucleotide pairs evaluated is 12, 11 and 13 in the *T. thermophilus*, *E. coli* I and *E. coli* II structures, after removal of observed crosslinks that have ≥14 Å between reactive bonds. For evaluation of H bonds/$s^4U$ and atom count around the $s^4U$, there are eight $s^4U$s that are involved in the 18 photocrosslinks that are evaluated in each of the three structures.

[5]The number of measurements of unreactive pairs is 311, 300 and 304 in the *T. thermophilus*, *E. coli* I and *E. coli* II structures respectively, except for the value of the $s^4U$ B factor, H bonds/$s^4U$, and atom count around the $s^4U$ which were evaluated just once on each of the substituted $s^4U$ positions in each structure.

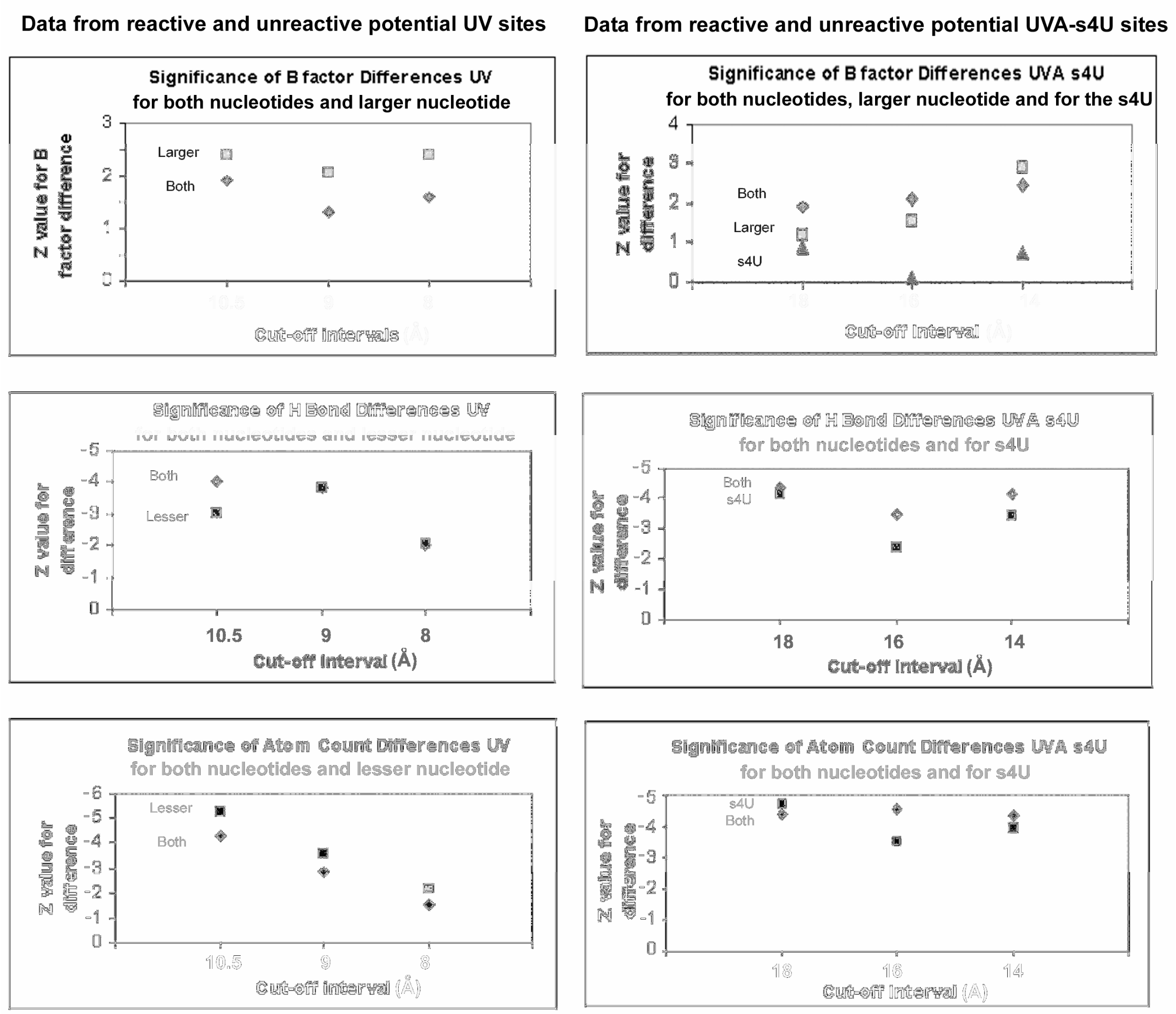


**Additional Figure 3. Plots of the z values for differences in internucleotide properties as a function of cut-off values for the internucleotide distance** Z values for the indicated comparisons summarized from Table 3 and Additional Tables 4 and 5 are plotted for the different internucleotide distance cut-off values. Differences are considered statistically significant if |z| is 1.96 or greater.

**Additional Table 8. Comparison of atom count around reactive and unreactive nucleotide pairs for different internucleotide distance intervals**

**UV reactions** — Nucleotide Pairs

| | | Reactive | | Unreactive | | | Statistics | |
|---|---|---|---|---|---|---|---|---|
| **Parameter** | **Dist. (Å)[1]** | **n[2]** | **Ave. ± S.D.** | **n** | **Ave. ± S.D.** | **Difference** | **z value** | **p value** |
| Atom count both nt. | 3-5 | 12 | 20.3 ± 8.3 | 63 | 18.5 ± 7.7 | 1.8 | 0.791 | 0.431 |
| | 5-6 | 4 | 17.9 ± 8.5 | 69 | 18.0 ± 9.2 | - 0.1 | -0.021 | 0.983 |
| | 6-7 | 12 | 20.1 ± 10.1 | 118 | 24.5 ± 9.3 | - 4.4 | -1.550 | 0.124 |
| | 7-8 | 4 | 28.0 ± 7.7 | 320 | 26.6 ± 9.1 | 1.4 | 0.306 | 0.760 |
| | 8-9 | 4 | 25.8 ± 10.8 | 504 | 28.7 ± 9.0 | - 2.9 | -0.641 | 0.552 |
| | 9-10 | 2 | 26.0 ± 10.8 | 589 | 29.7 ± 8.8 | - 3.7 | -0.593 | 0.552 |
| | 10-10.5 | 4 | 18.9 ± 10.2 | 496 | 30.0 ± 8.4 | -11.1 | -2.628 | 0.009 |
| Atom count lesser nt. | 3-5 | 12 | 15.2 ± 9.8 | 63 | 14.1 ± 7.3 | 1.1 | 0.466 | 0.642 |
| | 5-6 | 4 | 12.5 ± 10.2 | 69 | 13.2 ± 7.9 | - 1.3 | -0.170 | 0.865 |
| | 6-7 | 12 | 14.5 ± 7.5 | 118 | 18.8 8.3 | - 4.3 | -1.724 | 0.087 |
| | 7-8 | 4 | 21.8 ± 7.1 | 320 | 22.0 ± 9.2 | - 0.2 | -0.043 | 0.966 |
| | 8-9 | 4 | 19.3 ± 12.8 | 504 | 24.0 ± 9.3 | - 4.7 | -1.004 | 0.316 |
| | 9-10 | 2 | 23.0 ± 12.8 | 589 | 25.2 ± 8.8 | - 2.2 | -0.353 | 0.724 |
| | 10-10.5 | 4 | 6.8 ± 1.5 | 496 | 25.4 ± 8.7 | -18.6 | -4.271 | <0.0001 |

**UVA-s4U reactions** — Nucleotide Pairs

| | | Reactive | | Unreactive | | | Statistics | |
|---|---|---|---|---|---|---|---|---|
| **Parameter** | **Dist. (Å)[1]** | **n[2]** | **Ave. ± S.D.** | **n** | **Ave. ± S.D.** | **Difference** | **z value** | **p value** |
| Atom count both nt. | 3-6 | 16 | 20.7 ± 8.8 | 27 | 22.1 ± 9.3 | - 1.4 | -0.487 | 0.629 |
| | 6-9 | 5 | 24.7 ± 11.9 | 189 | 30.3 ± 8.1 | - 5.6 | -1.508 | 0.133 |
| | 9-11 | 3 | 25.3 ± 10.1 | 306 | 30.6 ± 8.0 | - 5.3 | -1.140 | 0.255 |
| | 11-13 | 5 | 20.6 ± 12.5 | 429 | 30.4 ± 9.1 | - 9.8 | -2.384 | 0.018 |
| | 13-15 | 11 | 29.2 ± 6.0 | 703 | 31.7 ± 7.8 | - 2.5 | -1.058 | 0.290 |
| | 15-17 | 7 | 24.2 ± 11.6 | 801 | 30.3 ± 8.9 | - 6.1 | -1.801 | 0.072 |
| | 17-18 | 3 | 23.9 ± 11.9 | 536 | 31.2 ± 8.1 | - 7.3 | -1.553 | 0.121 |
| Atom count lesser nt. | 3-6 | 16 | 14.8 ± 8.7 | 27 | 17.2 ± 8.3 | - 2.4 | -0.900 | 0.372 |
| | 6-9 | 5 | 15.2 ± 16.5 | 189 | 26.2 ± 8.4 | -11.0 | -2.808 | 0.006 |
| | 9-11 | 3 | 17.0 ± 10.0 | 306 | 25.9 ± 8.2 | - 8.9 | -1.868 | 0.063 |
| | 11-13 | 5 | 8.6 ± 8.6 | 429 | 25.5 ± 9.4 | -16.9 | -4.000 | <0.0001 |
| | 13-15 | 11 | 25.0 ± 4.3 | 703 | 27.5 ± 8.0 | - 2.5 | -1.034 | 0.302 |
| | 15-17 | 7 | 15.0 ± 12.1 | 801 | 25.5 ± 9.4 | -10.5 | -2.935 | 0.003 |
| | 17-18 | 3 | 13.2 ± 12.2 | 536 | 26.5 ± 8.4 | -13.2 | -2.729 | 0.007 |

[1]Nucleotide pairs were sorted into groups according to their internucleotide distance, measured between photoreactive bonds.

[2] n is the number of nucleotide pairs in each internucleotide distance interval. Reactive and unreactive nucleotide pair measurements were from *T. thermophilus* (11), *E. coli I* and *E. coli II* (13) structures.
